# Exogenous hCG activity, but not endogenous LH activity, is positively associated with live birth rates in anovulatory infertility

**DOI:** 10.3109/14647273.2011.587135

**Published:** 2011-07-07

**Authors:** Joan-Carles Arce, Johan Smitz

**Affiliations:** 1 Reproductive Health, Global Clinical & Non-Clinical R&D, Ferring Pharmaceuticals A/S, Copenhagen, Denmark; 2 Follicle Biology Laboratory and Center for Reproductive Medicine, Vrije Universiteit Brussel (VUB), Brussels, Belgium

**Keywords:** Highly purified menotrophin, human chorionic gonadotrophin, infertility, live birth, luteinising hormone, ovulation induction

## Abstract

**Objective:**

To evaluate, retrospectively, the roles of endogenous and exogenous luteinising hormone (LH) activity on live birth rate in ovulation induction cycles.

**Methods:**

Associations between LH activity at baseline, end of stimulation and live birth rate were analysed in relation to patient characteristics, baseline and end of stimulation variables in WHO group II anovulatory women (n=155) stimulated with recombinant follicle-stimulating hormone (rFSH) or highly purified human menopausal gonadotrophin (HP-hMG). HP-hMG provides FSH and exogenous LH activity mainly in the form of human chorionic gonadotrophin (hCG).

**Results:**

Serum LH concentrations at baseline or end of stimulation were not predictive of live birth rate in the rFSH group (n=79) or HP-hMG group (n=76). Serum hCG concentration at end of stimulation was a significant positive predictor in HP-hMG-treated women. Other variables were not independently predictive of live birth in either of the groups, except for a negative association between serum FSH concentrations at the start of stimulation and live birth in the rFSH-treated group.

**Conclusions:**

Endogenous LH concentrations are not predictive of live birth in anovulatory WHO group II patients undergoing ovulation induction with rFSH or HP-hMG. On the other hand, exogenous hCG activity during HP-hMG stimulation is positively associated with treatment outcome.

## Introduction

WHO group II anovulatory infertility is the most frequent anovulatory disorder, with the majority of such patients being diagnosed with polycystic ovary syndrome (PCOS) (van Santbrink et al., 1997; Laven et al., 2002). Management includes first-line treatment with clomiphene citrate (CC), and those who fail to ovulate or conceive during CC treatment respond well to gonadotrophin treatment (Hamilton-Fairley et al., 1991; Balen et al., 1994; Homburg & Howles, 1999).

Despite a normal follicle stimulating hormone (FSH) concentration, an elevated luteinising hormone (LH) concentration is not unusual in PCOS patients (Laven et al., 2002). Both the absolute concentration of circulating endogenous LH and the LH to FSH ratio are elevated in about 40% of these patients (Conway et al., 1989; Franks, 1989; Fauser et al., 1991; Balen et al., 1995; Taylor et al., 1997). The pathophysiology of the elevated LH concentration is not fully understood (Laven et al., 2002).

A number of studies are available on the impact of endogenous LH on fertility as well as treatment outcome during stimulated cycles in anovulatory patients. In women with regular spontaneous menstrual cycles, the endogenous LH concentration has been found to be higher in women with primary or secondary infertility than in fertile women (Regan et al., 1990). The association between elevated endogenous LH concentrations and miscarriage rates are inconsistent, both in women with normal ovarian morphology and in women with PCOS (Regan et al., 1990; Rai et al., 2000).

In PCOS women undergoing CC treatment, a high endogenous LH concentration *prior* to treatment has been associated with a high probability of conceiving (Kousta et al., 1997; Imani et al., 1999), whereas elevated endogenous LH concentrations *during* the follicular phase of CC treatment have been associated with poor treatment outcome (Shoham et al., 1990). An association between a high serum LH concentration in the follicular phase *during* gonadotrophin-releasing hormone (GnRH) pulsatile treatment and failure to conceive has been reported in PCOS patients (Jacobs & Homburg, 1990). A retrospective analysis in normogonadotrophic anovulatory patients (n= 154) suggested that the endogenous LH concentration prior to the start of ovulation induction with gonadotrophin preparations containing only FSH activity does not affect the chances of achieving a pregnancy (Mulders et al., 2003a). A few studies have measured LH concentrations during ovulation induction with FSH preparations (Coelingh Bennink et al., 1998; Balasch et al., 2003), but the impact of endogenous LH concentrations *during* or *at the end* of gonadotrophin stimulation on treatment outcome in PCOS patients has not been specifically addressed.

Due to the hypersecretion of LH in some patients with PCOS, gonadotrophin preparations containing only FSH activity were considered to have theoretical advantages over human menopausal gonadotrophin (hMG) preparations which contain both FSH and LH activity (Hillier, 1994; Shoham, 2002; Balasch, 2004). However, the existing data do not support these theoretical concerns, and there have been no studies powered to detect differences in clinical outcome. The available individual studies and meta-analyses have not been able to document significant differences in ongoing pregnancy and live birth rates between preparations containing only FSH activity and hMG preparations (Nugent et al., 2000; Platteau et al., 2006).

The major aim of the present retrospective study was to evaluate the roles of endogenous and exogenous LH activity on live birth rate in WHO group II anovulatory women stimulated with rFSH or highly purified menotrophins (HP-hMG), which provide FSH and exogenous LH activity mainly in the form of human chorionic gonadotrophin (hCG). Thus, serum concentrations of LH represent mainly the endogenous LH activity in both groups, and serum hCG represents the exogenous LH activity, which is present only in the HP-hMG group. Serum LH and hCG were compared between those women who achieved a live birth and those who did not, for both treatment regimens. To evaluate potential confounding predictors of live birth, we subsequently performed a full logistic regression analysis of the associations between live birth rate and an extensive range of patient characteristics, as well as endocrinological variables at baseline and end of stimulation.

## Material and methods

### Study population

This retrospective study was based on 184 women with normogonadotropic anovulatory infertility (WHO Group II) who participated in a prospective randomised controlled multicentre trial, which had ovulation rate as the primary outcome measure after ovulation induction with different gonadotrophin preparations [HP-hMG (Menopur, Ferring Pharmaceuticals A/S, Copenhagen, Denmark) and rFSH (Gonal-F, Merck Sereno, Geneva, Switzerland)] using a low-dose step-up protocol (Platteau et al., 2006). HP-hMG contributes both FSH and LH activity; the LH-activity being almost completely (>95% immunoreactivity) derived from hCG molecules (Wolfenson et al., 2005). As reported (Platteau et al., 2006), the ovulation rates in response to stimulation with the two gonadotrophin preparations are similar: 84.9% (79/93) for the rFSH group and 83.5% (76/91) for the HP-hMG group. In order to investigate the impact of a number of baseline and treatment-associated variables on live birth rate *per se* (i.e. ruling out the no live birth response due to lack of ovulation), only the women who ovulated after stimulation were included in the study cohort (n= 155). Main inclusion criteria were chronic anovulation (amenorrhoea, oligomenorrhea, or low progesterone concentrations in menstrual cycles of duration of 21-35 days), failure to ovulate with CC doses of at least 100 mg/day for at least 5 days or failure to conceive after three cycles of ovulation induction with CC, an age of at least 18 but not more than 39 years, a body mass index (BMI) of 19-35 kg/m^2^, and early follicular serum FSH concentrations between 1 and 12 IU/1. The study was carried out in accordance with the Declaration of Helsinki on good clinical practice, and ethical committee approval was obtained in all participating centres. Written informed consent was obtained from all trial subjects.

### Study protocol

Stimulation treatment was started 2-5 days after a spontaneous or progesterone-induced menstrual bleed. The starting dose of gonadotrophin was 75 IU daily, which was maintained for 7 days. After the first 7 days, the dose was either maintained or increased by 37.5 IU increments according to individual responses. All subjects were maintained on their specific dose concentration for at least 7 days. The maximum permitted daily dose was 225 IU, and the women were treated with the gonadotrophin for a maximum of 6 weeks. A single dose of 5,000 IU of hCG (Profasi, Merck Serono, Geneva, Switzerland) was given to trigger ovulation when one follicle of > 17 mm or two to three follicles of > 15 mm were observed by vaginal ultrasound. Any medication for luteal support (e.g. progesterone or hCG) was prohibited.

Blood samples were taken on stimulation day 1 (prior to the start dosing with gonadotrohins) and at the end of stimulation (at least 8h after the last gonadotrophin dose). Serum was analysed for endocrine variables by a central laboratory using an electrochemiluminescence immunoassay (LH, FSH, hCG, testosterone, prolactin, sex hormone-binding globulin (SHBG)), a radioimmunoassay (estradiol, androstenedione), a chemoluminescence assay (insulin), and an enzymatic method (glucose). The lower detection limits of the validated analytical methods were as follows: LH 0.10 IU/1, FSH 0.10 IU/1, hCG 0.10 IU/1, total testosterone 0.17 nmol/1, prolactin 0.3 (xg/1, SHBG 2 nmol/1, estradiol 55 pmol/1, androstenedione 0.10 nmol/1, insulin 14.4 pmol/1 and glucose 1.1 mmol/1.

Ovulation was defined as a serum progesterone concentration of ≥7.9 ng/ml (≥25 nmol/1) in the mid-luteal phase (6-9 days after hCG). Clinical pregnancy was defined as a transvaginal ultrasound showing at least one intrauterine gestation sac with foetal heart beat 7-9 weeks after hCG administration. Live birth was defined as a cycle that resulted in at least one live born neonate, regardless of the number of other neonates (live born or still born).

### Statistical analysis

Student's t-test was used for comparison of continuous variables between those women who did, and those who did not, achieve a live birth. The demographic and pre-stimulation variables as well as the variables obtained at end of ovarian stimulation for each treatment group were included in a primary univariate logistic regression analysis with the dependent variable live birth. The predictive value of each variable was summarised as an odds ratio with 95% confidence interval. The hCG values were multiplied by 10 to have a more interpretable OR estimate. All variables with a p-value <0.05 in the univariate regression analysis were then included in a secondary multivariate regression analysis for each treatment group. The multivariate model was reduced stepwise and the variables that were significant predictors of live birth at the <5% level are presented. Model fit was checked by the Hosmer and Lemeshow Goodness-of-Fit test. Tests for differences between variables in subgroups stratified into the 25th and 75th percentiles of exogenous serum hCG concentrations at end of stimulation were performed using the Kruskal-Wallis test for continuous data and the Chi-square test for categorical data.

## Results

Table I shows the demographics and baseline characteristics of the women who ovulated in response to stimulation. All prestimulation variables were comparable in the two treatment groups.

**Table I:** Demographics and baseline characteristics.

Variables	rFSH (n=79)	HP-hMG (n=76)	All (n= 155)
Female age (years)	29.4±3.8	29.4±4.0	29.4±3.9
BMI (kg/m^2^)	25.2±4.3	25.8±5.1	25.5±4.7
Waist-to-hip ratio	0.82±0.09	0.83±0.12	0.83±0.10
Primary infertility (%)	65	55	60
Duration of infertility (years)	2.9±1.9	2.9±1.8	2.9±1.9
Previous ovulation induction cycles	4.8±2.5	4.7±2.5	4.7±2.5
Clomiphene citrate non-responders			
Failure to ovulate* (%)	38	53	45
Failure to conceive† (%)	62	47	55
Mean ovarian volume (cm^3^)	8.2±4.3	8.1±4.1	8.1±4.2
Antral follicle count	22±14	23±17	23±16
LH (IU/1)	7.6±4.5	7.2±4.8	7.4±4.6
LH > 10 IU/1 (%)	23	22	23
FSH (IU/1)	5.4±2.7	5.1±1.3	5.2±2.1
LH:FSH ratio	1.6±1.2	1.5±1.1	1.5±1.1
Prolactin (μg/1)	12±7	13±15	12±11
Androstenedione (nmol/1)	7.2±3.5	8.0±5.0	7.6±4.3
Total testosterone (nmol/1)	1.6±0.6	1.8±0.7	1.7±0.6
SHBG (nmol/1)	62±43	57±38	59±40
Free androgen index	4.1±3.3	4.9±4.6	4.5±4.0
Estradiol (pmol/1)	158±63	156±91	157±77
Glucose (mmol/1)	5.1±0.7	5.2±0.7	5.1±0.7
Insulin (pmol/1)	98±101	109±117	103±109
Insulin:glucose ratio	2.8±2.6	2.9±2.7	2.8±2.7

Variables expressed as mean±SD, or %.

* At least 100 mg/day for at least 5 days.

† After three cycles.

Seventeen women in the rFSH group had a positive clinical pregnancy test and 16 of them achieved a live birth (14 singletons and 2 multiples). In the HP-hMG group, 14 women had a positive clinical pregnancy test and 13 of them achieved a live birth (all singletons). The endogenous LH concentrations prior to treatment did not differ significantly between the live birth and non-live birth subgroups in rFSH- or HP-hMG-treated women (Table II). Neither were the endogenous LH concentrations at end of stimulation significantly different between the two subgroups of each treatment regimen. However, the mean exogenous serum concentration of hCG at end of HP-hMG treatment was found to be 30% higher (p=0.010) in the subgroup of women who achieved a live birth compared with the non-live birth subgroup.

**Table II:** Serum concentrations of LH at baseline, and of LH and hCG at end of gonadotrophin stimulation.

	rFSH			HP-hMG		
		
LH activity	No live birth (n=63)	Live birth (n=16)	P	No live birth (n=63)	Live birth (n=13)	P
Baseline						
Endogenous LH (IU/1)	7.56±4.42	8.00±4.93	0.454	6.97±4.04	8.54±7.63	0.287
End of stimulation						
Endogenous LH (IU/1)	14.5±17.8*	9.41±6.54	0.289	11.2±9.91*	9.45±9.11	0.666
Exogenous hCG (IU/1)	-	-	-	0.98±0.35*	1.27±0.39	0.010

The data are expressed as mean±SD.

* 57 women attended the end-of-stimulation visit.

In the group of women who were stimulated with rFSH, the univariate logistic regression analysis of the associations between demographic and prestimulation variables as well as the variables obtained at end of ovarian stimulation and live birth showed that there was no significant association between the endogenous LH concentration and live birth rate; neither at baseline [OR=1.02 (95% CI: 0.91-1.15), p=0.723] nor at end of stimulation [OR=0.97 (95% CI: 0.92-1.02), p=0.273) (Table III). The only variable significantly associated with live birth rate in this treatment group was the serum concentration of FSH at baseline, which was negatively associated with live birth rate [OR=0.58 (95% CI: 0.38-0.90), p=0.015]. The variables yielding a p-value <0.1 in the univariate tests entered into a multivariate logistic regression analysis. As in the univariate tests, only FSH at baseline was a significant predictor of live birth rate at the 5% level.

**Table III:** Univariate logistic regression analysis of the association between live birth and clinical, sonographic and endocrinological parameters prior to start of ovarian stimulation and at end of stimulation.

	rFSH (n=79)			HP-hMG (n=76)		
		
Variables	OR	95% CI	p-value	OR	95% CI	p-value
Baseline						
Female age (years)	0.89	0.76-1.04	0.139	0.94	0.80-1.09	0.395
BMI (kg/m^2^)	1.07	0.94-1.21	0.318	0.96	0.85-1.08	0.508
Waist-to-hip ratio	1.02	0.55-1.88	0.944	1.32	0.84-2.05	0.226
Menstrual cycle pattern*	3.75	0.78-18.03	0.098	6.00	0.73-49.3	0.095
Duration of infertility (years)	0.90	0.64-1.27	0.551	0.70	0.41-1.19	0.188
Failure to ovulate on cc	0.54	0.18-1.63	0.271	0.65	0.19-2.19	0.482
Failure to conceive on cc	1.86	0.62-5.65	0.271	1.55	0.46-5.26	0.482
Antral follicle count	1.02	0.99-1.06	0.192	1.03	1.00-1.07	0.070
Mean ovarian volume (cm^3^)	1.02	0.90-1.15	0.761	0.99	0.85-1.16	0.948
LH (IU/1)	1.02	0.91-1.15	0.723	1.06	0.95-1.19	0.289
LH (≤10 IU/1 vs. >10 IU/1)	0.59	0.17-2.02	0.397	0.48	0.12-1.87	0.289
FSH (IU/1)	0.58	0.38-0.90	0.015	1.08	0.69-1.70	0.733
Estradiol (pmol/1)	1.00	0.99-1.01	0.993	0.99	0.97-1.00	0.041
Prolactin (μg/1)	0.97	0.88-1.07	0.516	0.92	0.80-1.05	0.207
Androstenedione (nmol/1)	1.07	0.92-1.25	0.357	0.94	0.80-1.10	0.443
Total testosterone (nmol/1)	1.96	0.77-4.95	0.155	0.56	0.20-1.53	0.255
SHBG (nmol/1)	1.00	0.99-1.01	0.863	1.01	0.99-1.02	0.481
Free androgen index	1.14	0.98-1.33	0.090	0.86	0.69-1.09	0.208
Glucose (mmol/1)	0.67	0.28-1.60	0.361	0.60	0.24-1.52	0.282
Insulin (pmol/1)	1.00	1.00-1.01	0.811	0.99	0.98-1.00	0.205
Insulin: glucose ratio	1.05	0.87-1.27	0.629	0.77	0.50-1.16	0.210
End of stimulation						
Follicular development (multiple vs. mono)	1.81	0.58-5.63	0.306	0.63	0.16-2.54	0.516
Duration of gonadotrophin (days)	1.04	0.93-1.17	0.507	1.02	0.93-1.12	0.675
Total dose of gonadotrophin (IU)	1.00	1.00-1.00	0.641	1.00	1.00-1.00	0.735
Endometrial thickness (mm)	1.25	0.98-1.61	0.078	0.90	0.67-1.20	0.461
Estradiol (pmol/1)	1.00	1.00-1.00	0.349	1.00	1.00-1.00	0.442
FSH (IU/1)	0.80	0.59-1.08	0.147	1.18	0.87-1.61	0.282
hCG (IU/1)	-	-	-	1.25	1.04-1.50	0.015
LH (IU/1)	0.97	0.92-1.02	0.273	0.98	0.91-1.05	0.568
LH (< 10 IU/1 vs. > 10 IU/1)	0.87	0.28-2.67	0.807	1.01	0.29-3.47	0.993

OR, odds ratio; CI, confidence interval.

* Amenorrhea and oligomenorrhea vs. anovulatory cycles.

In the univariate logistic regression analysis of the group of women stimulated with HP-hMG, two variables were found to be significantly associated with the live birth rate; the serum concentrations of estradiol at start of stimulation [OR=0.99 (95% CI: 0.97-1.00), p = 0.041] and exogenous hCG at end of stimulation [OR=1.25 (95% CI: 1.04-1.50), p = 0.015]. Similar to the rFSH group, neither the endogenous LH concentration at start of stimulation [OR=1.06 (95% CI: 0.95-1.19), p = 0.289] nor the LH concentration at end of stimulation [OR=0.98 (95% CI: 0.91-1.05), p = 0.568] was significantly associated with live birth rate. Furthermore, there was no correlation (p = 0.340) between the concentrations of LH and hCG at end of stimulation in the group of women stimulated with HP-hMG (data not shown). There was a trend towards an association between live birth rate and antral follicle count [OR=1.03 (95% CI: 1.00-1.07),p = 0.070], while no other demographic, baseline, clinical, sono-graphic or endocrine variable was associated with live birth rate. When eliminating the non-significant variables stepwise in a multivariate logistic regression analysis, the exogenous hCG concentration at the end of stimulation was the only significant predictor of live birth rate at the 5% level in the HP-hMG group. Model fit was checked by the Hosmer and Lemeshow Goodness-of-Fit test, which gave an acceptable fit (p = 0.518).

**Figure 1:**
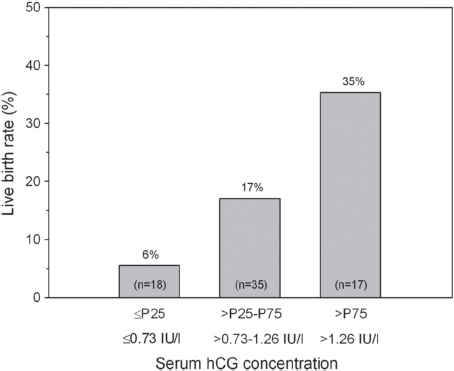
Live birth rate according to the ≤P25, >P25-P75 and >P75 percentiles of serum hCG concentrations at end of HP-hMG stimulation. Seventy women attended the end-of-stimulation visit.

The HP-hMG treated women were stratified into the 25th and 75th percentiles (P) of exogenous serum hCG concentrations at end of stimulation, resulting in subgroups of <P25, >P25-P75, and >P75. The live birth rate was lowest (6%) in the <P25 quartile and highest (35%) in the >P75 quartile (Figure 1). No significant differences between the percentile subgroups of hCG were observed regarding the demographic and baseline characteristics and the endocrine and sonographic variables at the start of stimulation, except for a significantly (p=0.028) higher antral follicle count in the >P25-P75 percentile compared to the two extreme quartiles and a trend (p=0.099) towards higher BMI in those women with lower hCG concentrations. Concerning stimulation characteristics, the duration of stimulation, the total dose of gonadotrophin and the dose on the last stimulation day as well as the levels of FSH and estradiol at end of stimulation were found to be highest in the >P75 quartile (Table IV).

**Table IV:** Demographics, baseline and stimulation characteristics by hCG concentration at end of HP-hMG stimulation.

	hCG (IU/1)			
		
	<P25	>P25-P75	>P75	
		
Variables	n=18	n=35	n=17	p-value*
Baseline				
Female age (years)	30.1±3.6	29.3±3.9	28.6±4.7	0.561
BMI (kg/m^2^)	27.8±4.1	26.0±5.5	24.3±5.3	0.099
Waist-to-hip ratio	0.83±0.06	0.83±0.12	0.86±0.17	0.759
Duration of infertility (years)	3.0±1.5	2.7±2.1	2.7±1.3	0.432
Primary infertility	39%	61%	53%	0.251
Antral follicle count	17±11	27±15	21±24	0.028
Mean ovarian volume (cm^3^)	8.2±3.5	8.4±4.2	7.3±4.5	0.468
LH (IU/1)	5.3±2.0	6.9±4.0	9.1±7.4	0.452
FSH (IU/1)	5.4±1.4	4.7±1.3	5.4±1.3	0.174
Estradiol (pmol/1)	132±40	176±114	151±78	0.237
Progesterone (nmol/1)	3.1±1.3	4.7±10.0	2.9±2.3	0.545
Prolactin (μg/1)	16.6±27.3	11.9±6.6	10.5±6.3	0.533
Androstenedione (nmol/1)	7.7±3.7	7.9±4.7	9.2±7.2	0.974
Total testosterone (nmol/1)	1.7±0.5	1.8±0.6	1.8±1.0	0.583
SHBG (nmol/1)	43.3±25.8	60.2±38.1	61.5±47.0	0.200
Glucose (mmol/1)	5.3±0.7	5.2±0.8	5.0±0.6	0.714
Insulin (pmol/1)	135±132	120±136	74±57	0.099
Insulin:glucose ratio	3.6±3.0	3.2±3.2	2.1±1.5	0.133
End of stimulation				
Subjects with monofollicular development (%)	67	69	59	0.466
Number of follicles ≥12 mm	2±1	2±2	2±2	0.780
Duration of gonadotrophin (days)	9.9±2.6	13.6±5.3	19.9±7.7	<0.001
Total dose of gonadotrophin (IU)	746±198	1225±669	2014±1102	<0.001
Last gonadotrophin dose (IU)	75±0	97.5±27.6	121.3±33.9	<0.001
Endometrial thickness (mm)	9.6±1.8	9.5±2.2	9.5±2.6	0.964
Estradiol (pmol/1)	831±247	1455±1524	1644±1552	0.017
FSH (IU/1)	8.4±2.5	9.1±1.2	11.6±0.90	<0.001
hCG (IU/1)	0.6±0.1	1.0±0.2	1.5±0.2	<0.001
LH (IU/1)	14.8±10.9	8.8±6.6	10.9±12.6	0.053
Subjects with LH > 10 IU/1 (%)	56	29	41	0.156

Variables expressed as mean±SD, or %.

* Kruskal-Wallis test for continuous and chi-square test for categorical data.

## Discussion

The present study did not find a significant association between serum concentrations of LH at baseline and live birth rate, neither in the rFSH group nor in the HP-hMG group. In line with this observation, the endogenous LH levels were similar in women who achieved a live birth and in those who did not. The lack of association between the LH level and outcome in the current study is in agreement with the results of a retrospective study in women with normogonadotropic anovulatory infertility resistant to CC-therapy treated with FSH activity gonadotrophin preparations (Mulders et al., 2003a), but in contrast to the meta-analysis of four studies in this patient category undergoing ovulation induction with FSH (Mulders et al., 2003b). The meta-analysis found a small, but significant, positive association between elevated basal LH levels and pregnancy rates. The present study, however, used a single sensitive LH assay applied to all study samples in a central laboratory, which may partly explain the different results.

The present study shows for the first time that increased serum concentrations of exogenous hCG at the end of stimulation were associated with higher live birth rates in normogonadotropic anovulatory infertile women treated with HP-hMG. Actually, among all the variables obtained at baseline or end of stimulation, the hCG concentration was the only variable predictive of treatment outcome in the multivariate logistic regression analysis. This is an interesting, novel finding in a PCOS population with already normal or high endogenous LH concentrations prior to starting the ovulation induction treatment. In this context, it is important to note that any interpretation of the relation between endogenous LH concentrations and outcome in HP-hMG-treated women is confounded by the fact that there is an intervention with exogenous hCG, supplementing additional LH activity.

The higher serum hCG concentrations (as well as FSH concentrations) in patients in the highest quartile group at the end of stimulation could be explained by their higher final daily dose of HP-hMG. These patients had a longer duration of stimulation in order to achieve the desired follicular response, but also had significantly higher estradiol levels than the patients in the other quartiles, despite an apparently comparable degree of follicular response. The finding of higher estradiol levels despite similar follicular response could reflect the effect of higher hCG levels on follicular differentiation. Also, the number of antral follicles was different between the percentile subgroups, but was not higher for the >75th percentile. A trend towards a lower BMI in the highest quartile group is in line with a previous report (Chan et al., 2003), which showed an inverse association between BMI and bioavailability of hCG. Despite all these observations, the concentration of hCG at the end of HP-hMG stimulation was identified as the sole significant positive predictor of live birth rate, while demographic variables, including BMI, or other variables at baseline or at end of stimulation did not add further to the prediction.

The interpretation of this investigation is influenced by certain methodological aspects of the hormone measurements. Immunoassay determination of LH concentrations based on blood sampling every 15 minutes leads to documented major oscillations within short intervals, which are attributed to the pulsatile release of LH and potentially to the exogenous pharmacological interventions (Griesinger & Diedrich, 2006). In the present study, the analysis of endogenous LH concentrations at baseline and the end of stimulation was based on a single blood sample on each occasion which may not truly reflect the mean daily endogenous LH concentration or the overall exposure of reproductive tissues to LH. Furthermore, the biological activity of the LH and hCG concentrations may not readily be extrapolated from the results of an immunoassay. Given the longer terminal half-life of hCG, the bioactivity contribution *in vivo* of hCG may be greater than could actually be extrapolated from immunoassay concentrations (de Leeuw et al., 1996).

As WHO type II anovulatory patients generally have normal or elevated LH concentrations, supplementation of LH activity is not considered to be required in this patient category. However, the finding in the present study that the live birth rate in the >75th percentile of hCG in HP-hMG stimulated women was 35% compared to 20% in the rFSH group may suggest that exogenous hCG exerts effects other than pure LH substitution. To date, no clinical studies have been adequately designed to investigate the effect of LH supplementation on live birth rates in anovulatory infertility WHO type II patients. Most of the data on potential favourable effects of LH activity on oocyte/embryo quality and on the endometrium are based on superovulating patients undergoing in vitro fertilisation/intracytoplasmic sperm injection (IVF/ICSI) cycles or oocyte donors, which usually exclude patients with PCOS (Acevedo et al., 2004; Platteau et al., 2004; Lisi et al., 2005; Smitz et al., 2007; Ziebe et al., 2007; Weghofer et al., 2008; Huddleston et al., 2009).

In summary, in anovulatory patients with normal or elevated baseline endogenous LH concentrations undergoing ovulation induction with gonadotrophins, the LH concentration at baseline or end of stimulation is not significantly associated with live birth rate. The exogenous LH activity, provided mainly in the form of hCG by using the HP-hMG gonadotrophin preparation, does not appear to be detrimental to live birth rates. Patients with higher serum hCG levels at the end of stimulation actually had the higher live birth rates in this treatment regimen.
